# Association of intensity of ventilation with 28-day mortality in COVID-19 patients with acute respiratory failure: insights from the PRoVENT-COVID study

**DOI:** 10.1186/s13054-021-03710-6

**Published:** 2021-08-06

**Authors:** Michiel T. U. Schuijt, Marcus J. Schultz, Frederique Paulus, Ary Serpa Neto, for the PRoVENT–COVID Collaborative Group, J. P. van Akkeren, A. G. Algera, C. K. Algoe, R. B. van Amstel, O. L. Baur, P. van de Berg, D. C. J. J. Bergmans, D. I. van den Bersselaar, F. A. Bertens, A. J. G. H. Bindels, M. M. de Boer, S.den Boer, L. S. Boers, M. Bogerd, L. D. J. Bos, M. Botta, J. S. Breel, H. de Bruin, S. de Bruin, C. L. Bruna, L. A. Buiteman-Kruizinga, O. Cremer, R. M. Determann, W. Dieperink, D. A. Dongelmans, H. S. Franke, M. S. Galek Aldridge, M. J. de Graaff, L. A. Hagens, J. J. Haringman, N. F. L. Heijnen, S. Hiel, S. T. van der Heide, P. L. J. van der Heiden, L. L. Hoeijmakers, L. Hol, M. W. Hollmann, M. E. Hoogendoorn, J. Horn, R. van der Horst, E. L. K. Ie, D. Ivanov, N. P. Juffermans, E. Kho, E. S. de Klerk, A. W. M. Koopman, M. Koopmans, S. Kucukcelebi, M. A. Kuiper, D. W. de Lange, D. M. van Meenen, Ignacio Martin-Loeches, Guido Mazzinari, N. van Mourik, S. G. Nijbroek, M. Onrust, E. A. N. Oostdijk, F. Paulus, C. J. Pennartz, J. Pillay, L. Pisani, I. M. Purmer, T. C. D. Rettig, J. P. Roozeman, M. T. U. Schuijt, M. J. Schultz, A. Serpa Neto, M. E. Sleeswijk, M. R. Smit, P. E. Spronk, W. Stilma, A. C. Strang, A. M. Tsonas, P. R. Tuinman, C. M. A. Valk, F. L. Veen, A. P. J. Vlaar, L. I. Veldhuis, P. van Velzen, W. H. van der Ven, P. van Vliet, P. van der Voort, H. H. van der Wier, L. van Welie, H. J. F. T. Wesselink, B. van Wijk, T. Winters, W. Y. Wong, A. R. H. van Zanten

**Affiliations:** 1 Department of Intensive Care, Amsterdam UMC, Location AMC, Meibergdreef 9, 1105 AZ Amsterdam, The Netherlands; 2 Mahidol Oxford Tropical Medicine Research Unit (MORU), Mahidol University, Bangkok, Thailand; 3 Nuffield Department of Medicine, University of Oxford, Oxford, UK; 4 ACHIEVE, Centre of Applied Research, Faculty of Health, Amsterdam University of Applied Sciences, Amsterdam, The Netherlands; 5 Department of Critical Care Medicine, Australian and New Zealand Intensive Care Research Centre (ANZIC-RC), Monash University, Melbourne, Australia

**Keywords:** Coronavirus disease 2019, COVID-19, Acute respiratory failure, ICU, Invasive ventilation, Driving pressure, ΔP, Mechanical power of ventilation, Mechanical power, Mortality

## Abstract

**Background:**

The intensity of ventilation, reflected by driving pressure (ΔP) and mechanical power (MP), has an association with outcome in invasively ventilated patients with or without acute respiratory distress syndrome (ARDS). It is uncertain if a similar association exists in coronavirus disease 2019 (COVID-19) patients with acute respiratory failure.

**Methods:**

We aimed to investigate the impact of intensity of ventilation on patient outcome. The PRoVENT-COVID study is a national multicenter observational study in COVID-19 patients receiving invasive ventilation. Ventilator parameters were collected a fixed time points on the first calendar day of invasive ventilation. Mean dynamic ΔP and MP were calculated for individual patients at time points without evidence of spontaneous breathing. A Cox proportional hazard model, and a double stratification analysis adjusted for confounders were used to estimate the independent associations of ΔP and MP with outcome. The primary endpoint was 28-day mortality.

**Results:**

In 825 patients included in this analysis, 28-day mortality was 27.5%. ΔP was not independently associated with mortality (HR 1.02 [95% confidence interval 0.88–1.18]; *P* = 0.750). MP, however, was independently associated with 28-day mortality (HR 1.17 [95% CI 1.01–1.36]; *P* = 0.031), and increasing quartiles of MP, stratified on comparable levels of ΔP, had higher risks of 28-day mortality (HR 1.15 [95% CI 1.01–1.30]; *P* = 0.028).

**Conclusions:**

In this cohort of critically ill invasively ventilated COVID-19 patients with acute respiratory failure, we show an independent association of MP, but not ΔP with 28-day mortality. MP could serve as one prognostic biomarker in addition to ΔP in these patients. Efforts aiming at limiting both ΔP and MP could translate in a better outcome.

*Trial registration* Clinicaltrials.gov (study identifier NCT04346342).

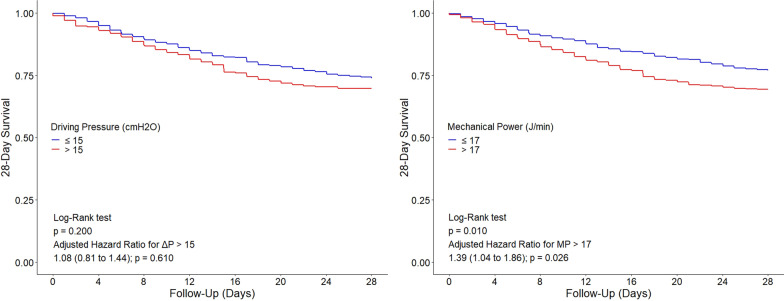

**Supplementary Information:**

The online version contains supplementary material available at 10.1186/s13054-021-03710-6.

## Introduction

Mortality rates are high in coronavirus disease 2019 (COVID-19) patients who need invasive ventilation for acute respiratory failure [1, 2]. Adequate prognostication is difficult though essential towards identifying patients with a high mortality risk, in order to consider alternative approaches hoping to improve outcomes. Studies have shown associations of several factors, like gender, age, comorbidities, biochemical markers and severity of illness scores with mortality [3–11]. Studies have also shown associations of various parameters of invasive ventilation, including degree of hypoxemia, positive end–expiratory pressure (PEEP), tidal volume (V_T_) and respiratory system compliance (Crs) with outcome in these patients [4, 5, 8–11].

In patients receiving ventilation because of a reason other than COVID-19, recent studies have shown associations of the driving pressure (ΔP) and the mechanical power of ventilation (MP) with mortality, both in patients with and patients without acute respiratory distress syndrome (ARDS) [12–20]. ΔP is the pressure applied by the ventilator used to deliver of a V_T_, as such representing the strain applied to the lung with each breath. MP is a summary value for the amount of energy per unit of time transferred from the ventilator to the respiratory system [15], and part of this energy acts directly on lung tissue, where it can cause harm. MP is calculated from V_T_, RR and the ΔP. These two parameters are attractive digital biomarkers, because they are easily calculable at the bedside, readily available and without costs.

Associations of ΔP and MP with outcome in COVID-19 patients that need invasive ventilation for acute respiratory failure have not yet been investigated. To investigate the impact of intensity of ventilation on 28-day mortality, we performed a preplanned analysis of a conveniently–sized national multicenter cohort of COVID-19 patients receiving invasive ventilation because of acute respiratory failure. The hypothesis was that both ΔP and MP have independent associations with mortality.

## Methods

### Design

This report concerns a preplanned secondary analysis of the PRoVENT-COVID study, an investigator–initiated, multicenter, retrospective observational study of invasively ventilated COVID-19 patients undertaken during the first 3 months of the pandemic at 22 ICUs in the Netherlands [8]. The study protocol of the PRoVENT-COVID study was prepublished [21], and a statistical analysis plan for the current analysis, written before assessing the database, is available online [22]. Details on the parent study have been published before [8]. The institutional review board of the Amsterdam UMC, Amsterdam, The Netherlands, approved the study protocol, and need for patient informed consent was waived seen the observational design of the study.

### Patients

Consecutive patients aged 18 years or older were eligible for participation in the PRoVENT-COVID study if admitted to one of the participating ICUs and had received invasive ventilation for acute respiratory failure due to COVID-19. The parent study had no exclusion criteria––for the current analysis we excluded patients with incomplete ventilation data to calculate ΔP or MP. We also excluded patients that were lost to follow–up at day 28.

### Collected data, patient classification, and calculations

Demographics and data regarding premorbid diseases and home medication were collected. On the first calendar day of invasive ventilation, in the first hour after intubation and thereafter every 8 h at fixed time points, ventilator settings and parameters were collected.

First, it was determined whether there was evidence of spontaneous breathing. Spontaneous breathing was deemed likely if: 1) patient was on a spontaneous ventilation mode, e.g., pressure support ventilation; or 2) patient was on a non–spontaneous ventilation mode with measured (total) RR exceeding the set RR > 2 breaths per minute. ΔP and MP were only calculated for those time points at which there was no evidence of spontaneous breathing. Per each time point, dynamic ΔP and MP were calculated using the following standard formulas:

1 $${\text{dynamic}}\,\Delta P\,\left( {{\text{in}}\,{\text{cm}}\,{\text{H}}_{2} {\text{O}}} \right) = {\text{peak}}\,{\text{pressure}}\,\left( {P_{{{\text{peak}}}} } \right){-}{\text{PEEP}}\quad \left[ {12,16} \right]$$

2 $${\text{MP}}\,\left( {{\text{in}}\,{\text{J}}/\min } \right) = 0.098*V_{T} *RR*\left( {P_{{{\text{peak}}}} {-}0.5*\Delta P} \right)\quad \left[ {15,16,23,24} \right]$$

The ΔP and MP were summarized as the mean of values over the first calendar day of ventilation.

### Outcomes

The primary outcome was 28-day mortality.

### Statistical analysis

Continuous variables were reported as median (quartile 25%–quartile 75%) and compared with Wilcoxon rank–sum tests, and categorical variables as number in percentage and compared with Fisher exact tests.

Variables with a *P* < 0.10 in the univariable prediction model were selected and included in the multivariable model. Variables with *P* < 0.05 in the multivariable model were selected as the covariates to be included in the final models. The following variables were considered for initial assessment: age, gender, body mass index, partial pressure of arterial oxygen/fraction of inspired oxygen (PaO_2_/FiO_2_) ratio, plasma creatinine, medical history of hypertension, heart failure, diabetes, chronic kidney disease, chronic obstructive pulmonary disease, active hematological neoplasia and/or active solid tumor, use of angiotensin converting enzyme inhibitors, use of angiotensin II receptor blockers, use of a vasopressor or inotropes, fluid balance, pH, mean arterial pressure, heart rate, and respiratory system compliance. These baseline covariates were selected according to clinical relevance and as used in previous study [8].

A multivariable (shared–frailty) Cox proportional hazard model including the covariates selected from those described above, and considering mean ΔP or MP as the predictor of interest was constructed. To compare the relative predictive ability of both variables, an additional model was build including ΔP and MP together, after assessing correlation and multicollinearity through Pearson’s correlation coefficient and variance inflation factor, respectively. If multicollinearity was found, this model was discarded and we followed with the independent models for ΔP and MP. For all models, the hazard ratio (HR) with its 95% confidence interval (CI) was reported. To further assess the impact of ΔP and MP, quintiles of increasing ΔP and MP were created, and the estimates for each quintile derived from the model above were plotted. Also, a double stratification analysis was used to assess the impact of each of the variables when the other was kept constant. First, the cohort was stratified in six quantiles of ΔP and then each quantile was further stratified in quartiles of increasing MP. The resulting quartiles have matched ΔP and increasing MP. Then, the models above were reproduced to extract the hazard ratio for each of the quartiles. Similarly, the same strategy was followed to create quartiles with matched MP and increasing ΔP.

Kaplan–Meier curves were used to compare 28-day mortality among patients receiving high and low mean ΔP and MP. The cutoff used for ΔP was set at 15 cm H2O [12, 16], though the ideal threshold for dynamic ΔP is less certain than for static ΔP. The ideal cutoff for MP has not yet been established, but recent reports suggest that a value of 17 J/min could be a reasonable cutoff for this parameter [13, 16, 25].

Two sensitivity analyses were performed. First, the models were re–ran according to the degree of hypoxemia at the first day of invasive ventilation. For this, we used the cutoffs as used in the Berlin definition for ARDS: mild (200 < PaO2/FiO2 ≤ 300 mmHg), moderate (100 < PaO2/FiO2 ≤ 200 mmHg) and severe hypoxemia (PaO2/FiO2 ≤ 100 mmHg). The models were repeated considering an interaction between the variable of interest (ΔP or MP) and the degree of hypoxemia at baseline. Second, the models were re–ran with an alternative validated equation for calculating MP in patients treated with pressure-controlled ventilation (PCV);

3 $${\text{MP}}_{{{\text{PCV}}}} \left( {{\text{in}}\,{\text{J}}/\min } \right) = 0.098*V_{T}*RR*\left( {\Delta P \, + {\text{ PEEP}}} \right)\quad [26].$$

All analyses were performed using R version 4.0.2 (R Foundation for Statistical Computing), and a *P* < 0.05 was considered significant.

## Results

### Patients

Of the originally enrolled 1102 patients in the PRoVENT-COVID study, 825 (74.9%) were used in the current analysis (Additional file 1: Figure S1). Demographics characteristics and ventilation characteristics are presented in Table 1. Most patients had moderate ARDS, using severity classification of the current Berlin definition for ARDS. The most prevalent premorbid conditions were hypertension and diabetes mellitus. 227 (27.5%) patients died within the first 28 days of follow–up. Other clinical outcomes are shown in Additional file 1: Table S1.

**Table 1:** Baseline characteristics and clinical outcome of the included patients according to the cohort studied

	Overall cohort (*n* = 825)
Age, years	65.0 (57.0–71.0)
Male gender – no (%)	600 (72.7)
Body mass index, kg/m^2^	27.8 (25.2–30.8)
Transferred under invasive ventilation	130 (15.8)
Days between intubation and admission	0.0 (0.0–0.0)
Use of non–invasive ventilation prior to intubation – no (%)	67/751 (8.9)
Duration of non–invasive ventilation, h	6.5 (2.0–19.9)
Chest CT scan performed – no (%)	268/799 (33.5)
Lung parenchyma affected – no (%)	
0%	13/268 (4.9)
25%	88/268 (32.8)
50%	78/268 (29.1)
75%	71/268 (26.5)
100%	18/268 (6.7)
Chest X–ray performed – no (%)	454/525 (86.5)
Quadrants affected – no (%)	
1	38/453 (8.4)
2	108/453 (23.8)
3	129/453 (28.5)
4	178/453 (39.3)
Severity of ARDS – no (%)	
No	9/813 (1.1)
Mild	73/813 (9.0)
Moderate	488/813 (60.0)
Severe	243/813 (29.9)
Co–existing disorders – no (%)	
Hypertension	279 (33.8)
Heart failure	35 (4.2)
Diabetes	191 (23.2)
Chronic kidney disease	37 (4.5)
Baseline creatinine, µmol/L*	78.0 (62.0–98.0)
Liver cirrhosis	3 (0.4)
Chronic obstructive pulmonary disease	68 (8.2)
Active hematological neoplasia	12 (1.5)
Active solid neoplasia	21 (2.5)
Neuromuscular disease	3 (0.4)
Immunosuppression	19 (2.3)
Previous medication – no (%)	
Systemic steroids	31 (3.8)
Inhalation steroids	92 (11.2)
Angiotensin converting enzyme inhibitor	142 (17.2)
Angiotensin II receptor blocker	90 (10.9)
Beta-blockers	149 (18.1)
Insulin	61 (7.4)
Metformin	135 (16.4)
Statins	251 (30.4)
Calcium channel blockers	157 (19.0)
Vital signs at day 01	
Heart rate, bpm**	85.0 (74.5–97.8)
Mean arterial pressure, mmHg**	80.5 (73.8–88.0)
Laboratory tests at day 01	
pH**	7.36 (7.31–7.41)
Worst PaO_2_/FiO_2_, mmHg***	123.9 (94.3–160.1)
PaCO_2_, mmHg**	44.5 (39.5–50.3)
Lactate mmol/L**	1.1 (0.9–1.4)
Organ support at day 01 – no (%)	
Continuous sedation	790/823 (96.0)
Inotropic or vasopressor	640/823 (77.8)
Vasopressor	639/823 (77.6)
Inotropic	41/823 (5.0)
Fluid balance, mL	539.0 (0.0–1340.0)
Urine output, mL	691.0 (380.0–1155.0)
Ventilation support at day 01	
Assisted ventilation – no (%)	151/823 (18.3)
Tidal volume, mL/kg PBW**^,a^	6.4 (5.9–7.0)
Tidal volume ≤ 8 mL/kg PBW	787 (96.1)
PEEP, cmH_2_O**^,a^	13.0 (11.0–14.7)
Peak pressure, cmH_2_O**^,a^	27.0 (24.2–30.0)
Driving pressure, cmH_2_O**^,a^	14.0 (12.0–16.0)
Driving pressure > 15 cmH_2_O	270 (32.7)
Mechanical power	
Absolute, J/min**^,a^	18.5 (15.5–22.2)
Mechanical power > 17 J/min	473 (57.3)
Adjusted by compliance, (J/min)/(mL/cmH_2_O)	0.57 (0.43–0.75)
Mechanical power > 0.23 (J/min)/(mL/cmH_2_O)	510 (61.8)
Compliance, mL/cmH_2_O**^,a^	32.1 (26.9–39.6)
Total respiratory rate, mpm**^,a^	21.7 (19.8–24.0)
Set respiratory rate, mpm**^,a^	22.0 (20.0–24.0)
Minute ventilation, L/min**^,a^	9.5 (8.4–11.0)
FiO_2_**	0.57 (0.48–0.68)
etCO_2_, mmHg**	36.9 (33.0–42.0)
Rescue therapy at day 01 – no (%)	
Prone positioning	263/811 (32.4)
Duration, h	8.0 (4.0–13.0)
Recruitment maneuver	14/667 (2.1)
ECMO	4/810 (0.5)
Use of NMBA	212/822 (25.8)
Hours of use	0.0 (0.0–8.0)
Clinical outcome	
28-day mortality	227 (27.5)

Data are median (quartile 25%–quartile 75%) or No (%). Percentages may not total 100 because of rounding

*CT* computed tomography; *ARDS* acute respiratory distress syndrome; *PaO2* arterial partial pressure of oxygen; *FiO2* Fraction of inspired oxygen; *PaCO2* arterial partial pressure of carbon dioxide; *PEEP* positive end expiratory pressure; *etCO2* End tidal carbon dioxide; *ECMO* extracorporeal membrane oxygenation; *NMBA* neuromuscular blocking agent

*Most recent measurement in 24 h before intubation, or at ICU admission under invasive ventilation

**Aggregate as the mean of a maximum of four values

***Worst value of four available

^a^Only assessed in moments without spontaneous breathing activity

### ΔP and MP

The median number of observations in the first calendar day of invasive ventilation at which ventilation data were collected was 3 [2 to 3]. In 88.2% [75 to 100%] of observations per patient, there was no evidence of spontaneous breathing and ΔP and MP could be calculated. Distribution of ventilator parameters are presented in Additional file 1: Figure S2.

In the first calendar day of invasive ventilation, median ΔP was 14.0 [12.0 to 16.0] cm H_2_O and median MP was 18.5 [15.5 to 22.2] J/min. ΔP was > 15 cm H_2_O in 270 (32.7%) patients; MP was > 17 J/min in 473 (57.3%) patients.

The baseline risk model used for the adjusted analysis is shown in Additional file 1: Table S2. The following variables were independently associated with outcome and selected as confounders for the final models: age, chronic obstructive pulmonary disease, pH and heart rate. No indication of multicollinearity between ΔP and MP was found in the model when including both variables together, thus, this model was not discarded (Additional file 1: Table S3).

### Association of ΔP and MP with 28-day mortality

**Fig. 1:**
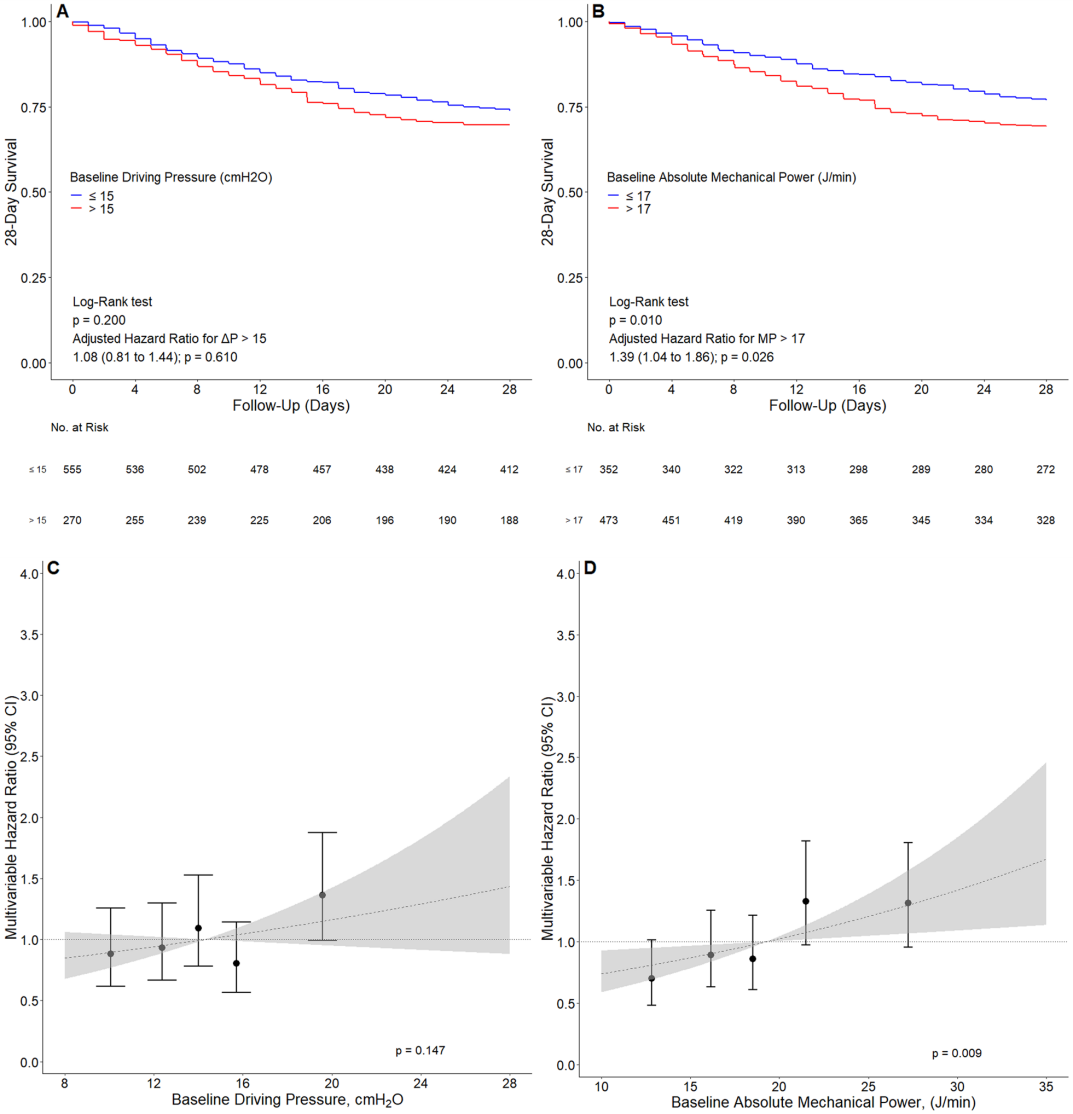
Association between driving pressure and mechanical power and 28-day mortality. **A** Kaplan–Meier curve comparing the 28-day mortality of patients ventilated with ΔP > 15 cmH_2_O vs. ΔP ≤ 15 cmH_2_O. **B** Kaplan–Meier curve comparing the 28-day mortality of patients ventilated with MP > 17 (J/min) versus ≤ 17 (J/min). **C** and **D** Effect of increasing levels of ΔP or MP on 28-day mortality. Circles and error bars are hazard ratio and 95% confidence interval for 5 quantiles of increasing ΔP or MP. Dashed lines and grey areas represent hazard ratio and 95% confidence interval for increasing values of ΔP or MP analyzed as a continuous variable and centralized in the mean of each variable. All models were adjusted for age, chronic obstructive pulmonary disease, pH, and heart rate. ΔP: driving pressure; MP: mechanical power

ΔP had no association with 28-day mortality, neither in the univariable (HR, 1.09 [95% CI, 0.96 to 1.24]; *P* = 0.190), nor in the multivariable assessment (HR, 1.02 [95% CI, 0.88 to 1.18]; *P* = 0.750) (Fig. 1 and Table 2). Contrary, MP had an association with 28-day mortality, both in an univariable (HR, 1.17 [95% CI, 1.02 to 1.33]; *P* = 0.020) and in a multivariable assessment (HR, 1.17 [95% CI, 1.01 to 1.36]; *P* = 0.031). While 28-day mortality was not different between patients with ΔP > 15 cm H_2_O versus ≤ 15 cm H_2_O, 28-day mortality was higher in patients with MP > 17 J/min versus ≤ 17 J/min (Fig. 1).

**Table 2:** Univariable and multivariable model assessing the association of baseline driving pressure and mechanical power with 28-day mortality

	Univariable model		Multivariable model for ΔP		Multivariable model for MP		Multivariable model for ΔP and MP	
	Hazard ratio (95% CI)	*p* value	Hazard ratio (95% CI)	*p* value	Hazard ratio (95% CI)	*p* value	Hazard ratio (95% CI)	*p* value
*Demographic characteristics*								
Age	1.87 (1.58–2.21)	< 0.001	1.89 (1.58–2.25)	< 0.001	1.91 (1.60–2.28)	< 0.001	1.91 (1.61–2.28)	< 0.001
*Co-existing disorders*								
COPD	1.81 (1.22–2.68)	0.003	1.70 (1.14–2.53)	0.009	1.78 (1.20–2.66)	0.004	1.79 (1.20–2.68)	0.004
*Laboratory tests at day 01*								
pH	0.68 (0.60–0.76)	< 0.001	0.75 (0.65–0.87)	< 0.001	0.77 (0.66–0.89)	< 0.001	0.77 (0.66–0.89)	< 0.001
*Vital signs at day 01*								
Heart rate	1.23 (1.08–1.41)	0.001	1.17 (1.01–1.35)	0.031	1.16 (1.01–1.34)	0.035	1.16 (1.01–1.34)	0.037
*Ventilatory variables at day 01*								
Driving pressure	1.09 (0.96–1.24)	0.190	1.10 (0.97–1.24)	0.147	–	–	1.02 (0.88–1.18)	0.750
Absolute mechanical power	1.17 (1.02–1.33)	0.020	–	–	1.18 (1.04–1.35)	0.009	1.17 (1.01–1.36)	0.031

Continuous variables were included and the hazard ratio represents the increase in one standard deviation of the variable

*ΔP* driving pressure; *MP* mechanical power; *CI* confidence interval; *COPD* chronic obstructive pulmonary disease

**Fig. 2:**
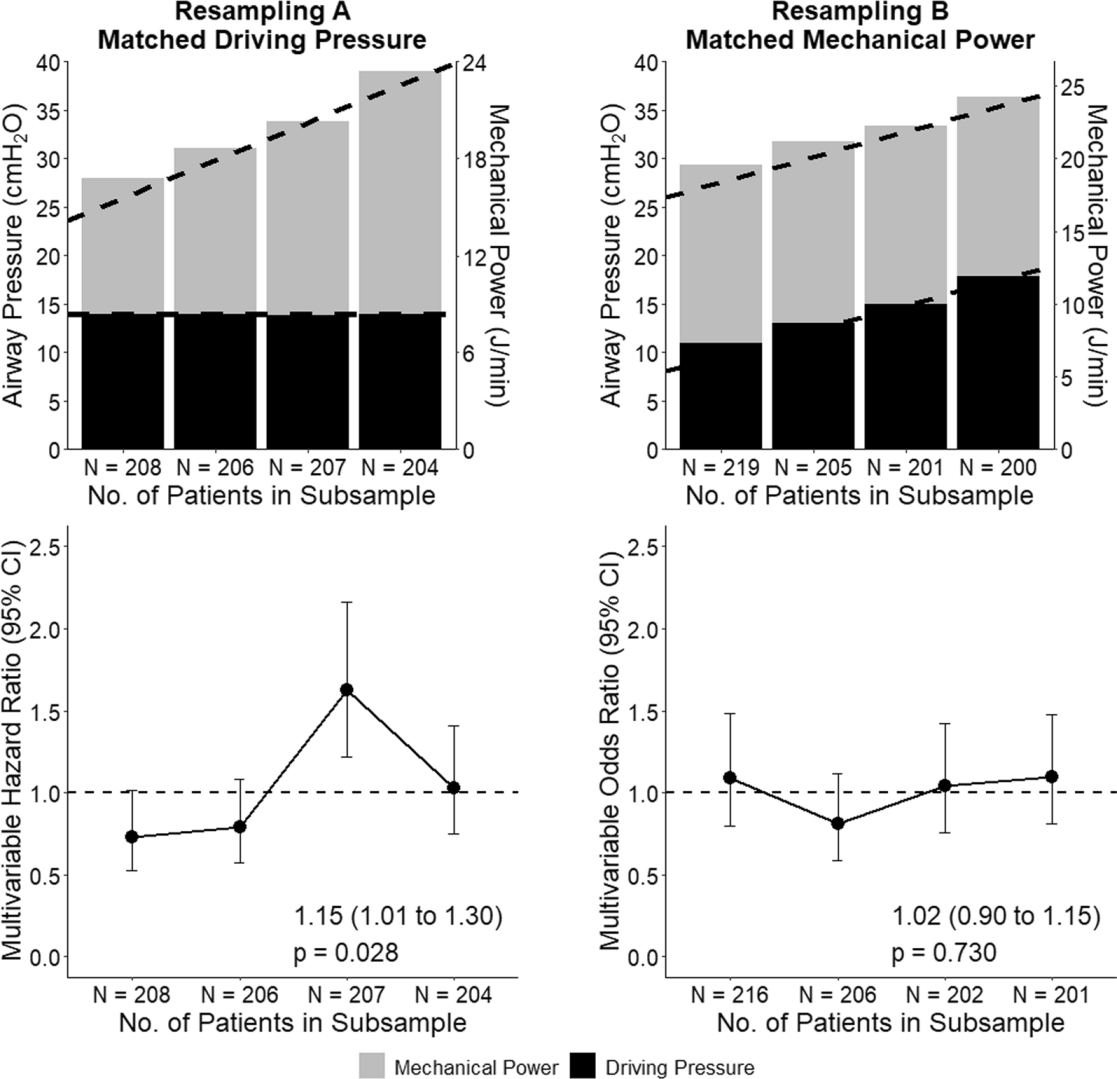
Hazard ratio for 28-day mortality across relevant quartiles of driving pressure and mechanical power. **A** (left) Comparable values of ΔP, but increasing values of MP across strata. Multivariable HR for increasing MP per stratum is presented below. **B** (right) Comparable values of MP, but increasing values of ΔP across strata. Multivariable HR for increasing ΔP per stratum is presented below. The Y1 axis is for airway pressure; the Y2 axis is for mechanical power. X axis reports cohort sample sizes. Circles and error bars in the lower panels are HR and 95% confidence interval for quartiles of increasing ΔP and matched MP or vice-versa. All models were adjusted for age, chronic obstructive pulmonary disease, pH, and heart rate

If ΔP was kept constant and only MP increased (i.e., due to increases in other components than ΔP) a statistically significant effect on outcome was found––increasing quartiles of MP, stratified on comparable levels of ΔP, were associated with increased risk of 28-day mortality (HR, 1.15 [95% CI, 1.01 to 1.30]; *P* = 0.028) (Fig. 2). However, increasing quartiles of ΔP, stratified on comparable levels of MP, were not associated with 28-day mortality (HR, 1.02 [95% CI, 0.90 to 1.15]; *P* = 0.730). No interaction between the effect of ΔP or MP on 28-day mortality and the degree of hypoxemia at baseline was found (Table 3). The sensitivity analysis using an alternative equation for MP in patients under PCV did not change the findings (Additional file 1: Table S4, Figure S2 and S3).

**Table 3:** Effect of driving pressure and mechanical power on 28-day mortality according to severity of hypoxemia at baseline

	Multivariable hazard ratio (95% CI)	*P* value
*Driving pressure*		
Mild ARDS	1.66 (0.97–2.84)	Reference
Moderate ARDS	1.01 (0.85–1.19)	0.083
Severe ARDS	1.12 (0.89–1.40)	0.173
*Mechanical power*		
Mild ARDS	1.10 (0.59–2.05)	Reference
Moderate ARDS	1.11 (0.94–1.29)	0.899
Severe ARDS	1.30 (1.01–1.68)	0.635

P value for the interaction between severity of ARDS and the variable of interest

*CI* confidence interval

## Discussion

In this observational study assessing the association of ΔP and MP on 28-day mortality of patients receiving invasive ventilation for acute respiratory failure related to COVID-19, higher ΔP was not, but higher MP was associated with increased 28-day mortality after adjustment for confounders. In addition, when ΔP was kept constant, progressive increments in MP, due to increase in other components, like V_T_ or RR, resulted in higher risks for 28-day mortality.

Comparing our patient cohort to COVID-19 patients in series of patients worldwide, baseline characteristics and 28-day mortality were similar [1, 11, 27–29]. Two large observational studies, originating from France and from the United States, reported a similar median ΔP, respectively 13 [10 to 17] and 15 [11 to 18] cm H_2_O. Thus far, MP has only been reported in one cohort of COVID-19 patient [11], in which MP was much higher than in our cohort, 26.5 [18.6 to 34.9] versus 18.5 [15.5 to 22.2] J/min. It remains uncertain why we see this remarkable difference, as the same equation for MP was used. However, MP in our study was comparable to that reported in cohorts of patients with ARDS from another origin than COVID-19 [13, 16, 19].

This is the first study on associations of ΔP and MP with mortality in invasively ventilated COVID-19 patients. In contrast to previous studies on ΔP and MP in patients receiving ventilation because of a reason other than COVID-19, higher ΔP was not associated with an increased risk of mortality. MP adjusted for confounders was associated with 28-day mortality, being in line with previous studies [13, 16, 19]. Furthermore, increasing quartiles of MP, stratified on comparable levels of ΔP, were associated with increased risk of 28-day mortality, showing the predictive value of MP in addition to ΔP, which is in line with a previous study in patients receiving ventilation because of a reason other than COVID-19 [18]. Recently, a study showed the adverse effect of the exposure to higher intensities of ΔP and MP over time in critically ill patients receiving ventilation due to respiratory failure due to a reason other than COVID-19 [16].

In our analysis, the signal for mechanical power was stronger than for ΔP. Over recent years, ΔP has become a value targeted by the clinician not to exceed a certain value. This may be caused by the fact that mechanical power is more difficult to calculate at the bedside than ΔP. Consequently, this may have resulted in lower ΔP levels with a narrow distribution in the current cohort, and this may have led to insufficient statistical power to test whether ΔP has a statistical association with outcome. In our cohort, MP was often high and with a broad distribution.

Despite the finding that the association of ΔP with 28-day mortality did not reach statistical significance, ΔP remains an important digital biomarker. Limiting ΔP has been found to have a strong potential to improve outcome in other patient cohorts [13, 14, 16]. Besides, ΔP is much easier to calculate at the bedside compared to MP. In daily practice, MP may serve as an additional digital biomarker that is calculated by, and presented on the screen of the ventilator. Nevertheless, randomized controlled trials evidence remains needed to understand the true and independent value of limiting ΔP and MP.

Various equations for calculating MP have been studied and reported in recent years [16–20, 25, 30]. As transpulmonary pressures and plateau pressures were not routinely measured, we used dynamic driving pressure in the MP equation. Recent findings suggest that this substitution is reliable [30], and other validated this approach [16]. Aside, using the dynamic ΔP simplifies the calculation of MP at the bedside. The sensitivity analysis, using another previously validated equation for MP in patients under PCV [26] did not change the findings.

Our study has several strengths. The study was conveniently–sized, and included a large number of centers. Also, both academic and non–academic centers participated, improving the generalizability of our findings. Granular ventilation data were collected by trained study personnel. We restricted the analysis to patients without the evidence of spontaneous breathing, as both ΔP and MP cannot yet be calculated in a reliable way in patients with spontaneous breathing, and we adhered to a prepublished statistical analysis plan.

Our study also has limitations. In this study we did not collect blood biomarkers, like D–dimer levels, which have been shown to have a strong association with mortality [10]. Therefore, we could not add them to our models. Likewise, pulmonary embolism was not included in our models, which could be a confounding factor, as increased death space could result in a higher MP. Furthermore, the disease severity scores were not included, as the participating centers used different scores, which are not mutually exclusive. However, multiple baseline covariates were used in our models, representing multiple organ systems, supportive treatments and pre–existing comorbidities, being in line with previous studies investigating the impact of ΔP and MP [16, 18, 19]. Also, during the first half of 2020, there was no standard use of dexamethasone or tocilizumab, which may influence patient outcome. Another limitation is that normalization of MP by respiratory system compliance or predicted body weight has shown superior predictive value over non–normalized MP [17]. However, normalized MP has been less validated in comparison to absolute MP. Also, it is unknown whether this relationship simply reflects more an association between respiratory system compliance and patient outcome.

## Conclusion

In this cohort of COVID-19 patients that received invasive ventilation for acute respiratory failure, both a higher MP and increasing quartiles of MP stratified on comparable levels of ΔP were associated with increased risk of 28-day mortality. Taken together, both ΔP and MP are useful digital biomarkers for prognostication in invasively ventilated COVID-19 patients. Targeting lower MP, in addition to lower ΔP, may translate in better outcomes.

## Supplementary Information

**Additional file 1. Table S1**. Additional Clinical Outcomes in the Included Cohort.** Table S2**. Univariable and Multivariable Model of Covariates Selected for Inclusion in the Final Models.** Table S3**. Multivariable Model Assessing the Association of Driving Pressure and Mechanical Power with 28–Day Mortality in the Same Model.** Table S4**. Univariable and Multivariable Model Assessing the Association of Baseline Mechanical Power Calculated for Patients Under PCV* with 28-Day Mortality.** Figure S1**. Flowchart of Included Patients.** Figure S2**. Ranges of ventilator variables.** Figure S3**. Association Between Mechanical Power for PCV and 28-Day Mortality.** Figure S4**. Hazard Ratio for 28–Day Mortality Across Relevant Quartiles of Driving Pressure and Mechanical Power for PCV.

## Data Availability

A dataset will be made available upon request to the corresponding authors one year after the publication of this study. The request must include a statistical analysis plan.

## Abbreviations

ARDS: Acute respiratory distress disease
CI: Confidence interval
C_RS_: Respiratory system compliance
COVID-19: Coronavirus disease 2019
ΔP: Driving pressure
ECMO: Extracorporeal membrane oxygenation
FiO_2_: Fraction of inspired oxygen
HR: Hazard ratio
ICU: Intensive care unit
MP: Mechanical power of ventilation
NMBA: Neuromuscular blocking agents
PaCO_2_: Partial pressure of arterial carbon dioxide
PaO_2_: Partial pressure of arterial oxygen
P_peak_: Peak pressure
PEEP: Positive end-expiratory pressure
PRoVENT-COVID: PRactice of VENTilation in COVID-19
RR: Respiratory rate
V_T_: Tidal volume
VFD-28: Ventilator-free days and alive at day 28
