# Supplementary material for: Association of intensity of ventilation with 28-day mortality in COVID-19 patients with acute respiratory failure: insights from the PRoVENT-COVID study
Source: Crit Care. 2021 Aug 6;25:283. doi: 10.1186/s13054-021-03710-6 (PMC8343355; doi:10.1186/s13054-021-03710-6)
Supplement: Supplementary file 1 — Additional file 1. Table S1. Additional Clinical Outcomes in the Included Cohort. Table S2. Univariable and Multivariable Model of Covariates Selected for Inclusion in the Final Models. Table S3. Multivariable Model Assessing the Association of Driving Pressure and Mechanical Power with 28–Day Mortality in the Same Model. Table S4. Univariable and Multivariable Model Assessing the Association of Baseline Mechanical Power Calculated for Patients Under PCV* with 28-Day Mortality. Figure S1. Flowchart of Included Patients. Figure S2. Ranges of ventilator variables. Figure S3. Association Between Mechanical Power for PCV and 28-Day Mortality. Figure S4. Hazard Ratio for 28–Day Mortality Across Relevant Quartiles of Driving Pressure and Mechanical Power for PCV. [file 13054_2021_3710_MOESM1_ESM.docx]

**Association of Intensity of Ventilation with 28–day Mortality in COVID–19 Patients with Acute Respiratory Failure – insights from the PRoVENT–COVID study**

| **eTable 1 – Additional Clinical Outcomes in the Included Cohort** | |
| --- | --- |
|  | **Overall Cohort**  **(*n* = 825)** |
| Ventilator–free days at day 28, days | 3.0 (0.0 – 16.5) |
| Duration of ventilation, days | 14.0 (8.0 – 23.0) |
| In survivors at day 28, days | 15.0 (9.0 – 27.3) |
| Tracheostomy – no (%) | 136 (16.6) |
| Thromboembolic complications – no (%) | 236 (28.6) |
| Pulmonary embolism | 184 (22.3) |
| Deep vein thrombosis | 42 (5.1) |
| Ischemic stroke | 27 (3.3) |
| Myocardial infarction | 11 (1.3) |
| Systemic arterial embolism | 4 (0.5) |
| Acute kidney injury – no (%) | 370 (45.1) |
| Need for RRT – no (%) | 147 (17.8) |
| Need of rescue therapy – no (%)* | 615 (75.3) |
| Prone positioning | 496 (60.5) |
| Recruitment maneuver | 38 (5.6) |
| Use of NMBA | 385 (46.7) |
| ECMO | 6 (0.7) |
| ICU length of stay, days | 15.0 (9.0 – 27.0) |
| In survivors, days | 17.0 (10.0 – 29.0) |
| Hospital length of stay, days | 23.0 (14.0 – 37.0) |
| In survivors, days | 29.0 (20.0 – 44.0) |
| ICU mortality – no (%) | 256 (31.4) |
| Hospital mortality – no (%) | 264 (34.6) |
| 90–day mortality – no (%) | 277 (36.4) |
| Data are median (quartile 25% – quartile 75%) or No (%). Percentages may not total 100 because of rounding  *RRT: renal replacement therapy; NMBA: neuromuscular blocking agent; ECMO: extracorporeal membrane oxygenation; ICU: intensive care unit*  * assessed in the first four days of ventilation | |

| **eTable 2 – Univariable and Multivariable Model of Covariates Selected for Inclusion in the Final Models** | | | | |
| --- | --- | --- | --- | --- |
|  | **Univariable Model** | | **Multivariable Model** | |
|  | **Hazard Ratio**  **(95% CI)** | ***p* value** | **Hazard Ratio**  **(95% CI)** | ***p* value** |
| **Demographic characteristics** |  |  |  |  |
| Age | 1.87 (1.58–2.21) | < 0.001 | **1.82 (1.52–2.19)** | **< 0.001** |
| Male gender | 1.15 (0.85–1.56) | 0.368 | ––– | ––– |
| Body mass index | 0.92 (0.80–1.06) | 0.247 | ––– | ––– |
| **Co–existing disorders** |  |  |  |  |
| Hypertension | 1.53 (1.17–1.99) | 0.002 | 1.21 (0.90–1.64) | 0.203 |
| Heart failure | 1.75 (1.01–3.03) | 0.046 | 1.23 (0.70–2.17) | 0.461 |
| Diabetes | 1.55 (1.16–2.07) | 0.003 | 1.32 (0.97–1.80) | 0.080 |
| Chronic kidney disease | 1.28 (0.73–2.24) | 0.393 | ––– | ––– |
| Chronic obstructive pulmonary disease | 1.81 (1.22–2.68) | 0.003 | **1.67 (1.12–2.50)** | **0.012** |
| Hematological neoplasia | 1.85 (0.75–4.57) | 0.179 | ––– | ––– |
| Solid tumor | 1.41 (0.65–3.02) | 0.383 | ––– | ––– |
| **Previous medication** |  |  |  |  |
| Angiotensin converting enzyme inhibitor | 1.32 (0.95–1.82) | 0.095 | 0.91 (0.64–1.30) | 0.614 |
| Angiotensin II receptor blocker | 1.14 (0.76–1.70) | 0.538 | ––– | ––– |
| **Organ support at day 01** |  |  |  |  |
| Use of inotropic or vasopressor | 1.16 (0.83–1.62) | 0.397 | ––– | ––– |
| Fluid balance | 1.13 (0.99–1.29) | 0.073 | 1.05 (0.91–1.20) | 0.491 |
| **Ventilation support at day 01** |  |  |  |  |
| Compliance | 0.94 (0.82–1.08) | 0.404 | ––– | ––– |
| **Laboratory tests at day 01** |  |  |  |  |
| PaO_2_ / FiO_2_ | 0.83 (0.71–0.97) | 0.017 | 0.87 (0.74–1.02) | 0.097 |
| Creatinine | 1.07 (0.99–1.15) | 0.094 | 1.04 (0.94–1.15) | 0.473 |
| pH | 0.68 (0.60–0.76) | < 0.001 | **0.77 (0.66–0.90)** | **0.001** |
| **Vital signs at day 01** |  |  |  |  |
| Mean arterial pressure | 0.88 (0.77–1.01) | 0.061 | 0.90 (0.79–1.03) | 0.142 |
| Heart rate | 1.23 (1.08–1.41) | 0.001 | **1.18 (1.02–1.36)** | **0.007** |
| *CI: confidence interval; PaO2: arterial partial pressure of oxygen; FiO2: fraction of inspired oxygen*  Continuous variables were included after standardization and the hazard ratio represents the increase in one standard deviation of the variable.  Variables with a *p* < 0.100 were selected for inclusion in the multivariable model and variables with *p* < 0.050 in the multivariable model (highlighted in bold) were selected for inclusion in the final model. | | | | |

| **eTable 3 – Multivariable Model Assessing the Association of Driving Pressure and Mechanical Power with 28–Day Mortality in the Same Model** | | | |
| --- | --- | --- | --- |
|  | **Hazard Ratio**  **(95% CI)** | ***p* value** | **VIF** |
| **Demographic characteristics** |  |  |  |
| Age | 1.91 (1.61–2.28) | < 0.001 | 1.032 |
| **Co–existing disorders** |  |  |  |
| Chronic obstructive pulmonary disease | 1.79 (1.20–2.68) | 0.004 | 1.033 |
| **Laboratory tests at day 01** |  |  |  |
| pH | 0.77 (0.66–0.89) | < 0.001 | 1.268 |
| **Vital signs at day 01** |  |  |  |
| Heart rate | 1.16 (1.01–1.34) | 0.037 | 1.245 |
| **Ventilatory variables at day 01** |  |  |  |
| Driving pressure | 1.02 (0.88–1.18) | 0.750 | 1.367 |
| Mechanical power | 1.17 (1.01–1.36) | 0.031 | 1.382 |
| *VIF: variance inflation factor*  Continuous variables were included after standardization and the hazard ratio represents the increase in one standard deviation of the variable.  Variance inflation factor > 2.50 indicates potential multicollinearity. | | | |

| **eTable 4 - Univariable and Multivariable Model Assessing the Association of Baseline Mechanical Power Calculated for Patients Under PCV* with 28-Day Mortality** | | | | |
| --- | --- | --- | --- | --- |
|  | **Univariable Model** | | **Multivariable Model for MP_PCV_** | |
|  | **Hazard Ratio**  **(95% CI)** | ***p* value** | **Hazard Ratio**  **(95% CI)** | ***p* value** |
| **Demographic characteristics** |  |  |  |  |
| Age | 1.87 (1.58–2.21) | < 0.001 | 1.92 (1.61–2.29) | < 0.001 |
| **Co-existing disorders** |  |  |  |  |
| Chronic obstructive pulmonary disease | 1.81 (1.22–2.68) | 0.003 | 1.73 (1.15–2.60) | 0.008 |
| **Laboratory tests at day 01** |  |  |  |  |
| pH | 0.68 (0.60–0.76) | < 0.001 | 0.78 (0.67–0.91) | 0.001 |
| **Vital signs at day 01** |  |  |  |  |
| Heart rate | 1.23 (1.08–1.41) | 0.001 | 1.17 (1.01–1.35) | 0.031 |
| **Ventilatory variables at day 01** |  |  |  |  |
| Absolute mechanical power for PCV | 1.14 (1.00 to 1.30) | 0.042 | 1.18 (1.03–1.35) | 0.016 |
| *ΔP: driving pressure; MP: mechanical power; CI: confidence interval; PCV: pressure-controlled ventilation*  *** Calculated as 0.098 * tidal volume in liters * respiratory rate * (driving pressure + PEEP)  Continuous variables were included and the hazard ratio represents the increase in one standard deviation of the variable. | | | | |

**eFigure 1 – Flowchart of Included Patients**

**
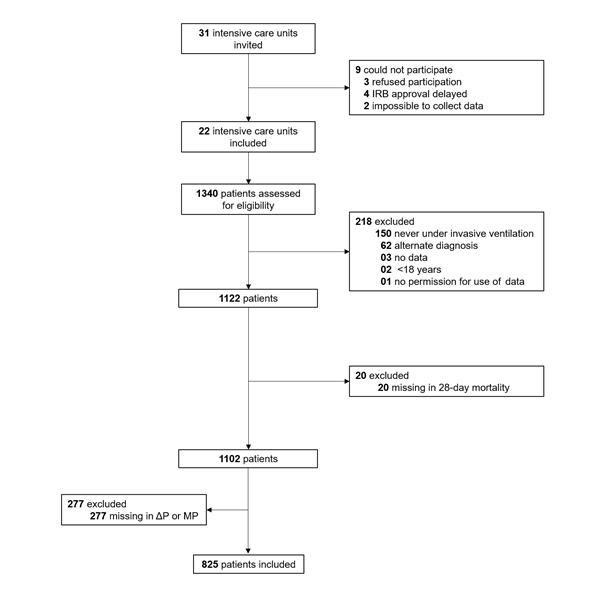
**

*ΔP: driving pressure; MP: mechanical power; IRB: institutional review board*

Missing in ΔP or MP could be due–the absence of moments without spontaneous activity during the period of interest.

**eFigure 2 – Ranges of ventilator variables**


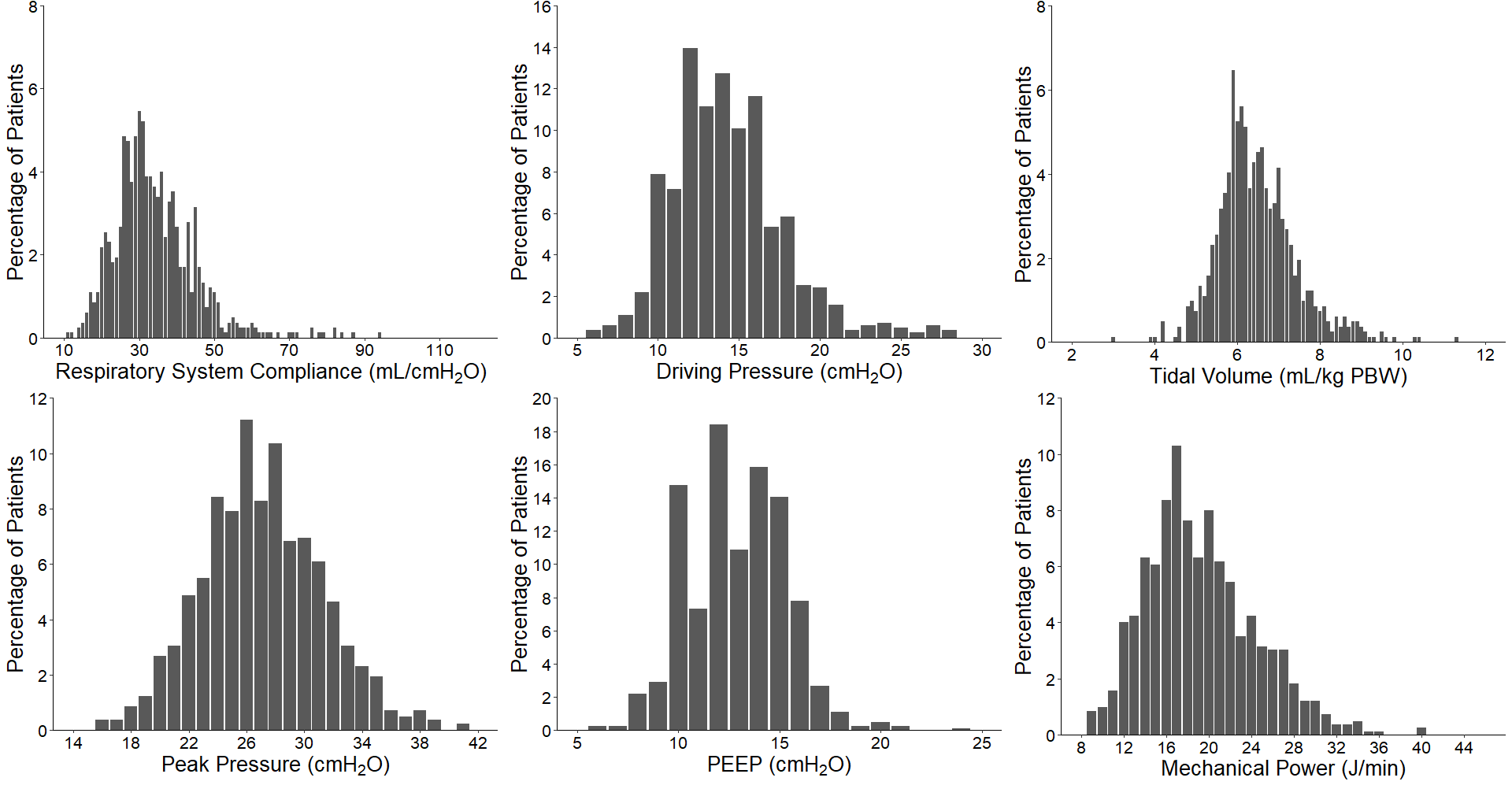


*PEEP: Positive end-expiratory pressure; PBW: predicted body weigth*

**eFigure 3 – Association Between Mechanical Power for PCV and 28-Day Mortality**

**
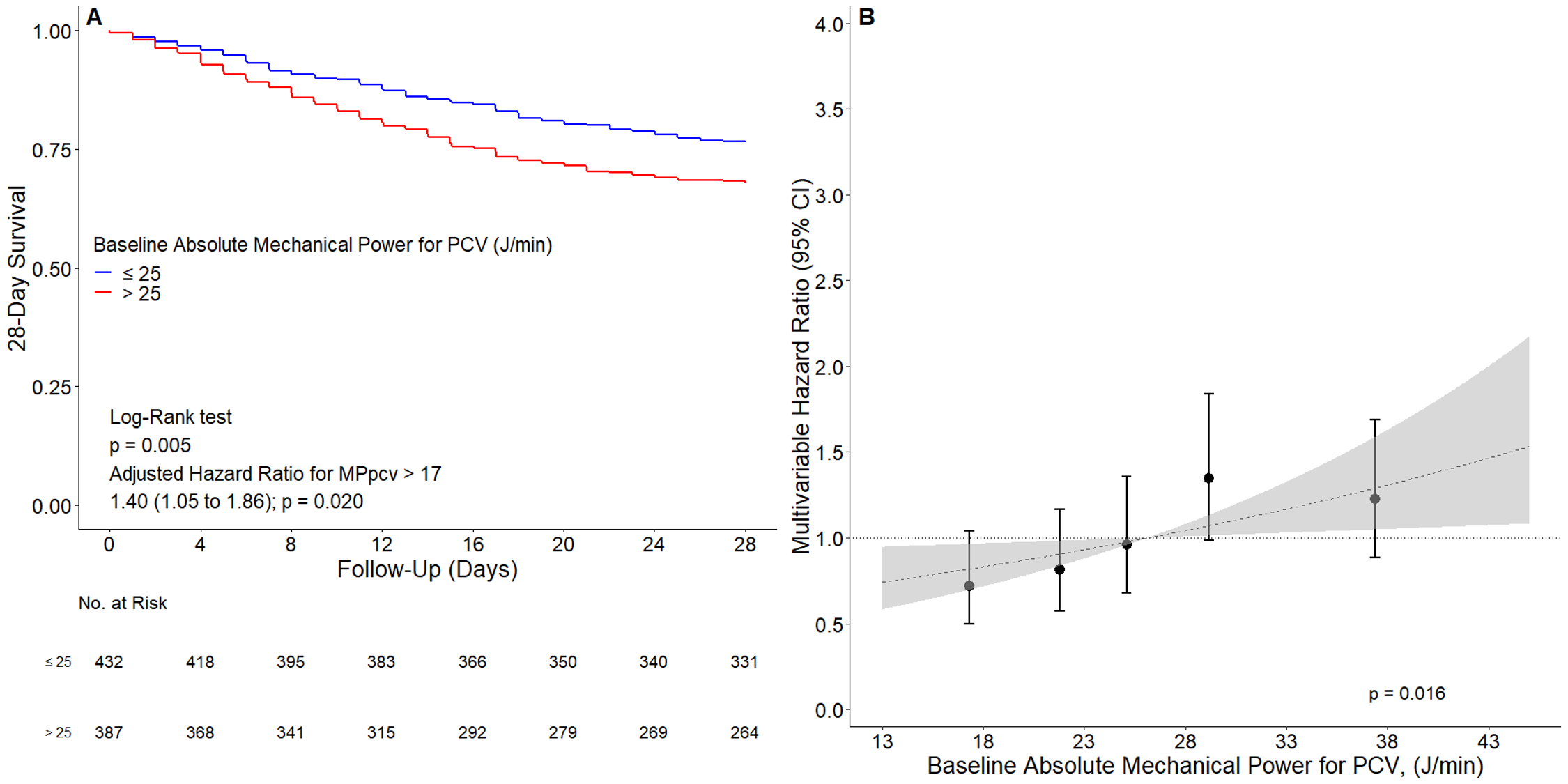
**

A, Kaplan–Meier curve comparing the 28–day mortality of patients ventilated with MP > 25 (J/min) versus ≤ 25 (J/min) (the median as used). B, Effect of increasing levels of MP for PCV on 28–day mortality. Circles and error bars are hazard ratio and 95% confidence interval for 5 quantiles of increasing MP for PCV. Dashed lines and grey areas represent hazard ratio and 95% confidence interval for increasing values of MP for PCV analyzed as a continuous variable and centralized in the mean of each variable.

All models were adjusted for age, chronic obstructive pulmonary disease, pH, and heart rate.

*MPpcv: mechanical power for pressure-controlled ventilation*

**eFigure 4 – Hazard Ratio for 28–Day Mortality Across Relevant Quartiles of Driving Pressure and Mechanical Power for PCV**

**
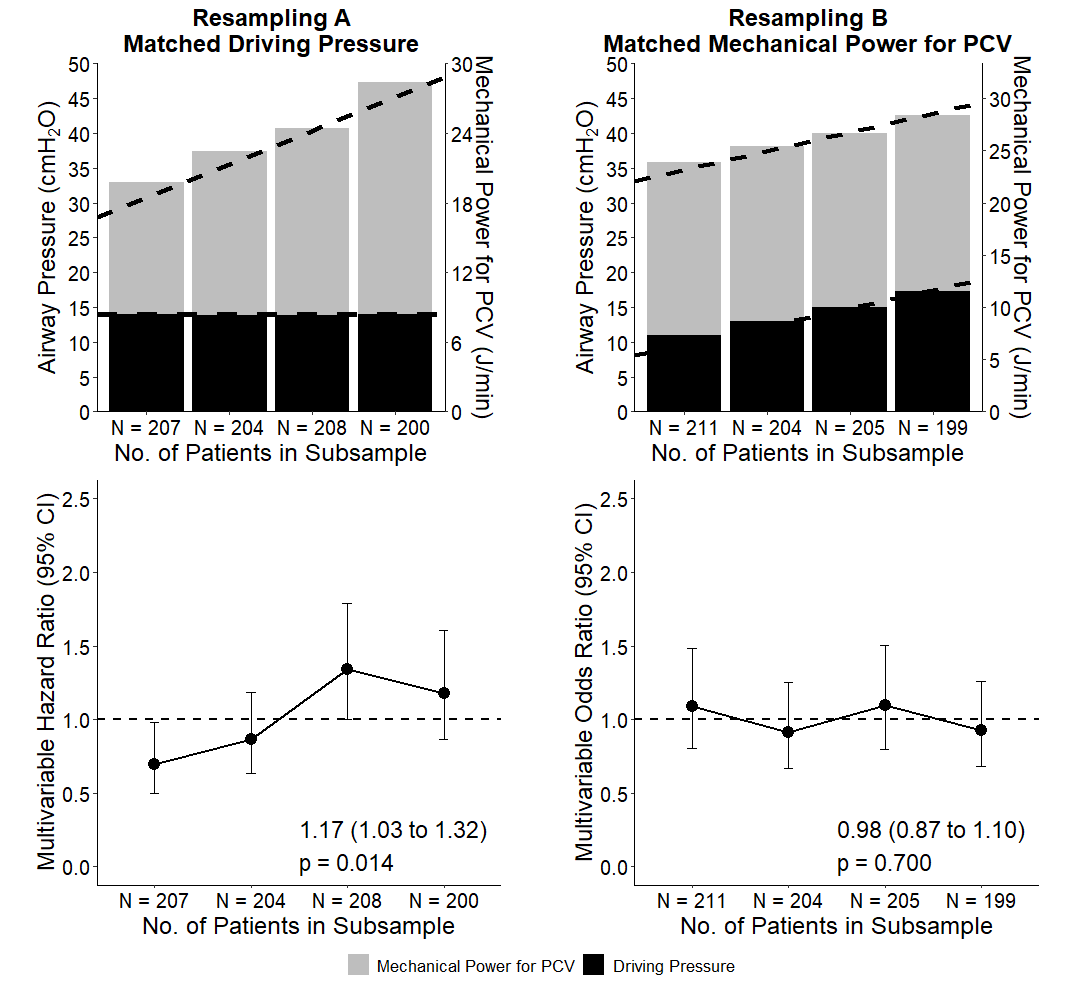
**

A (upper), Comparable values of ΔP, but increasing values of mechanical power for PCV across strata. HR for each stratum is presented below.

B (upper), Has comparable values of mechanical power for PCV, but increasing values of driving pressure across strata.

Y1 axis is airway pressure; Y2 axis is mechanical power for PCV. X axis reports cohort sample sizes. Circles and error bars are hazard ratio and 95% confidence interval for quartiles of increasing ΔP and matched MP for PCV *or* vice-versa. All models were adjusted for age, chronic obstructive pulmonary disease, pH, and heart rate.

*PCV: pressure-controlled ventilation*
